# Effect of perioperative FLOT *versus* ECF/ECX on short-term outcomes after surgery for resectable oesophagogastric adenocarcinoma: propensity score-matched study

**DOI:** 10.1093/bjsopen/zrac003

**Published:** 2022-02-23

**Authors:** Osama Moussa, Ricky Harminder Bhogal, George Malietzis, Charlotte Fribbens, Naureen Starling, Marco Gerlinger, David Watkins, Ian Chau, Sheela Rao, David Cunningham, William H. Allum, Asif Chaudry, Sacheen Kumar

**Affiliations:** 1 Division of Surgery & Cancer, Imperial College London, St Mary’s Hospital, London, UK; 2 Gastrointestinal Unit, The Royal Marsden Hospital, 203 Fulham Road, London, UK; 3 Upper GI Surgical Oncology Research Group, Division of Radiotherapy & Imaging, Institute of Cancer Research, London, UK; 4 Division of Molecular Pathology, Institute of Cancer Research, London, UK

## Abstract

**Background:**

Perioperative FLOT (fluorouracil plus leucovorin, oxaliplatin, and docetaxel) chemotherapy is a recent regimen used to treat resectable oesophagogastric (OG) adenocarcinoma, associated with improved overall survival *versus* earlier chemotherapy strategies. This study compared short-term perioperative morbidity in a large tertiary centre series of FLOT to a matched cohort receiving ECX/ECF (epirubicin, cisplatin, capecitabine (X) or 5-fluorouracil (F)).

**Methods:**

Consecutive patients completing four perioperative cycles of FLOT and proceeding to surgery with resectable OG adenocarcinoma were included. This was matched to patients from a historic ECX/ECF cohort from the same institution. A propensity score was calculated, and a secondary analysis using a propensity-matched group performed.

**Results:**

Cohorts were matched by tumour location and operations performed. In total there were 129 (64.5 per cent) oesophageal and 71 (35.5 per cent) gastric resections (FLOT 57 oesophageal, 43 gastric; ECF/ECX 64 oesophageal, 36 gastric). The median length of stay after surgery was 12 days in the FLOT group *versus* 15 in ECF/ECX (*P* = 0.035). There were no significant differences in overall perioperative complications and, specifically, no difference in OG anastomotic leaks, analysed by site (gastric (FLOT 0/79 (0 per cent) *versus* ECX 2/79 (2.5 per cent); *P* = 0.123), oesophageal (FLOT 4/121 (3.3 per cent) *versus* ECX 5/121 (4.1 per cent); *P* = 0.868) or type of surgery (open FLOT 1/121 (0.8 per cent) *versus* ECX 3/121 (2.5 per cent); *P* = 0.368; minimally invasive (FLOT 3/121 (2.5 per cent) *versus* ECX 2/121 (1.7 per cent); *P* = 0.555)). There was no statistical difference in leak-related return to theatre, 30-day (FLOT 0 (0 per cent) *versus* ECX 3/100 (3.0 per cent); *P* = 0.081), or 90-day (FLOT 0 (0 per cent) *versus* ECX 2/100 (2.0 per cent); *P* = 0.155) mortality.

**Conclusion:**

In terms of surgical complications, FLOT and ECX/ECF were equally safe in patients undergoing resection for OG adenocarcinoma.

## Teaser

A case-matched study comparing the perioperative outcomes of oesophagogastric adenocarcinoma in a single tertiary centre. There were no significant reported differences in perioperative complications between cohorts across all systemic classifications of benchmark complications.

## Introduction

Disease stage at diagnosis determines the outcome in patients with gastric or lower oesophageal cancer. Many patients present at a late stage, where distant metastases preclude treatment with curative intent. Few Western patients who present with early disease confined to the mucosa are suitable for surgery alone or endoscopic resection^1^. Most patients considered for surgery have locoregional disease, involving invasion within the wall and/or locoregional lymph node spread. Poor survival after surgery alone has led to a number of trials exploring the role of chemotherapy in addition to surgery^2–4^.

Several studies showed that chemotherapy was effective for these cancers in patients with advanced disease. The regimen of epirubicin, cisplatin, and infused fluorouracil (ECF) achieved a favourable response in locally advanced gastric^5,6^ and oesophagogastric (OG) cancer^7^, resulting in its use alongside surgery.

The MAGIC trial of perioperative chemotherapy for gastric and OG junctional adenocarcinoma combined three 3-week cycles of ECF before and after surgery, and demonstrated an improvement in 5-year overall survival *versus* surgery alone (36 per cent *versus* 23 per cent)^8^. In the French FNCLCC/FFCD 9703·3 study, patients received 2–3 cycles of cisplatin with fluorouracil before and after surgery or surgery alone, resulting in a significant and similar improvement in 5-year overall survival in the perioperative chemotherapy arm (38 per cent *versus* 24 per cent)^9^.

Recent evidence has supported a new regimen consisting of a triplet of 5-fluorouracil (5-FU), folinic acid, oxaliplatin, and docetaxel (FLOT). The FLOT4 trial compared perioperative therapy with FLOT with ECF/ECX (where oral capecitabine (X) was substituted in some patients for infusional 5-FU (F)). In locally advanced, resectable gastric or OG junctional adenocarcinoma, perioperative FLOT improved overall survival *versus* perioperative ECF/ECX (45 per cent *versus* 36 per cent)^10^.

There is limited evidence of the safety profile of FLOT and its impact on surgical complications after oesophageal and gastric cancer resection. This study investigated the perioperative outcomes of patients undergoing resection after completing four cycles of FLOT at a single tertiary OG cancer centre in the UK compared with a historic cohort of patients treated with preoperative ECF/ECX identified from the institutional database.

## Methods

The study included all patients with adenocarcinomas of the stomach, OG junction, and lower third of the oesophagus, who underwent and completed four cycles of preoperative FLOT and subsequently underwent oesophageal/gastric resection. Patients receiving other neoadjuvant chemotherapy regimens, undergoing a robotic minimally invasive procedure, those who underwent neoadjuvant chemotherapy outside this institution, and patients recruited to ongoing immunotherapy trials (ICONIC; NCT03399071), including FLOT chemotherapy, were excluded.

### Patient cohort

All FLOT patients from October 2017 (when FLOT was included in the institutional protocol) until March 2020 were identified from a prospectively developed database and compared with a similar number of patients treated between 2006 and 2017 with ECX/ECF. The perioperative outcomes for all patients were evaluated using the Esophageal Complications Consensus Group standardized platform for reporting complications, quality measures, and mortality associated with all OG resections^11^.

### Statistical analysis

Outcomes were tabulated in a similar format, and the data for the study were extracted, prepared, and analysed using SPSS version 25 (IBM, Armonk, New York, USA). Baseline demographic, clinical, and operative factors were compared across cohorts using Pearson’s χ^2^ test for categorical variables and Mann–Whitney or Kruskal–Wallis test for continuous data. All analyses were standardized using a binary logistic regression to standardize both groups according to sex, age group, BMI group, and performance status for operative details, perioperative morbidities, and histological outcomes. The authors used the Gastric & Oesophageal Cancer Staging American Joint Committee on Cancer, eighth edition^12,13^. The Mandard Regression system was also used for histological reporting^14^, and the Clavien-Dindo scoring criteria for estimating complications^15^.

To endorse matching the FLOT group to the ECF/ECX group, a propensity score was calculated for each patient with the main variables known to affect the outcome (age, sex, BMI, ASA, tumour site). For the propensity score, only complete data with no missing values were used. Patients with FLOT were matched to the closest patient with ECF at a 1:1 ratio to obtain comparable groups. All *P*-values reported were two-sided and statistical significance was considered when *P* < 0.05.

## Results

Patients treated with FLOT and ECF/ECX were similar in terms of age, sex, BMI, and performance status (*Table 1*). There were no differences in preoperative morbidities with respect to diabetes (13 per cent *versus* 10 per cent; *P* = 0.506), cardiac (19 per cent *versus* 18 per cent; *P* = 0.856), pulmonary (20 per cent *versus* 17 per cent; *P* = 0.585); and chronic renal disease (4 per cent *versus* 2 per cent; *P* = 0.407).

**Table 1:** Characteristics of both perioperative chemotherapy cohorts comparing both FLOT and ECF/ECX group baseline demographics and morbidities

Variable		FLOT (*n* = 100)	ECF/ECX (*n* = 100)	*P*
**Sex**	Male	78 (48.1)	84 (51.9)	0.279
	Female	22 (57.9)	16 (42.1)	
**ASA grade**	1	9 (47.4)	10 (52.6)	0.950
	2	64 (50.8)	62 (49.2)	
	3	27 (49.1)	28 (50.9)	
**WHO performance status score**	0	26 (41.3)	37 (58.7)	0.112
	1	63 (52.1)	58 (47.9)	
	2	11 (68.8)	5 (31.3)	
**Diabetes**	No	87 (49.2)	90 (50.8)	0.506
	Yes	13 (56.5)	10 (43.5)	
**Cardiac disease**	No	81 (49.7)	82 (50.3)	0.856
	Yes	19 (51.4)	18 (48.6)	
**Chronic pulmonary disease**	No	80 (49.1)	83 (50.9)	0.585
	Yes	20 (54.1)	17 (45.9)	
**Chronic renal disease**	No	96 (49.5)	98 (50.5)	0.407
	Yes	4 (66.7)	2 (33.3)	
**BMI category (kg/m^2^)**	< 20	7 (87.5)	1 (12.5)	0.112
	20–25	38 (52.1)	35 (47.9)	
	25–30	32 (43.2)	42 (56.8)	
	> 30	23 (51.1)	22 (48.9)	
**Age category**	< 40	4 (50.0)	4 (50.0)	0.146
	40–50	14 (77.8)	4 (22.2)	
	50–60	17 (43.6)	22 (56.4)	
	60–70	38 (50.7)	37 (49.3)	
	> 70	27 (45.0)	33 (54.0)	

Data are presented as *n* (%). FLOT, fluorouracil plus leucovorin, oxaliplatin, and docetaxel chemotherapy; ECF/ECX, (epirubicin, cisplatin, capecitabine (X) or 5-fluorouracil (F)).

The tumour characteristics of the two groups are shown in (*Table 2*). There was no difference in anatomical location between the groups, gastric (FLOT 43/79 (54.4 per cent) *versus* ECX 36/79 (45.6 per cent); *P* = 0.311) and oesophageal (FLOT 57/121 (47.1 per cent) *versus* ECX 64/121 (52.9 per cent); *P* = 0.311) (*Tables S1* and *S2*).

**Table 2:** Postoperative histopathological characteristics comparing the perioperative FLOT and ECF/ECX cohorts

Variable		FLOT (*n* = 100)	ECF/ECX (*n* = 100)	*P*
**Resection**	R0	95 (95)	86 (86)	0.030
	R1	5 (5)	14 (14)	
**Tumour location**	Oesophageal (total)	57 (47)	64 (3)	0.311
	Distal oesophageal	20 (67)	10 (33)	
	GOJ Siewert I	23 (41)	33 (59)	
	GOJ Siewert II	14 (40)	21 (60)	
	Gastric (total)	43 (54)	36 (46)	0.311
	GOJ Siewert III	10 (48)	11 (52)	
	Gastric body	15 (79)	4 (21)	
	Incisura	3 (37)	5 (62)	
	Pyloric/antral	15 (48)	16 (52)	
**T**	0	17 (17)	5 (5)	0.048
	1	16 (16)	14 (14)	
	2	13 (13)	19 (19)	
	3	45 (45)	56 (56)	
	4	9 (60)	6 (40)	
**N**	0	60 (60)	52 (52)	0.512
	1	20 (20)	28 (18)	
	2	11 (11)	9 (9)	
	3	9 (9)	11 (11)	
**Mandard response**	1 (no residual cancer)	20 (67)	10 (33)	0.062
	2 (rare residual cancer cells)	15 (54)	13 (46)	
	3 (fibrosis outgrowing residual cancer)	33 (52)	31 (48)	
	4 (residual cancer outgrowing fibrosis)	11 (31)	25 (69)	
	5 (absence of regressive changes)	21 (50)	21 (50)	
**Complete regression**		20 (20)	10 (10)	0.048

Data are presented as *n* (%). FLOT, fluorouracil plus leucovorin, oxaliplatin, and docetaxel chemotherapy; ECF/ECX, (epirubicin, cisplatin, capecitabine (X) or 5-fluorouracil (F)); GOJ, gastro-oesophageal junction.

Pathological T stage^12^ demonstrated a significance in tumour T stage regression favouring the FLOT cohort compared with the ECX cohort (*P* = 0.048), but there was no difference in nodal relapse (FLOT 60/100 (60 per cent) *versus* ECX 52/100 (52 per cent); *P* = 0.512). There was no difference in total lymph node harvest, with a median of 33 (IQR 23 to 43) in the FLOT group and 36 (IQR 26 to 45) in the ECF/ECX group (*P* = 0.262) (*Table S3*). There was a significant difference in the rate of positive microscopic circumferential resection margins (FLOT 5/100 (5 per cent) *versus* ECX 14/100 (14 per cent); *P* = 0.030). There were no involved longitudinal margins.

More patients exhibited complete tumour regression in the FLOT cohort (OR = 0.213; *P *= 0.018) on binary logistic regression when adjusting for age, sex, BMI, WHO status, and operation. There was no difference in cohorts with regard to grades of gastric staging (*P* = 0.426) in both groups, and in oesophageal staging (*P* = 0.070) using the eighth AJCC staging classification^12,13^. More patients underwent a minimally invasive oesophageal resection in the FLOT group (minimally invasive: FLOT 48/121 (84.2 per cent) *versus* ECF/ECX 26/121 (40.6 per cent); *P* < 0.001), but there was no difference in approach for gastric resections as all were performed via an open approach.

There was no difference in the leak rates after gastric resections (FLOT 0/79 (0 per cent) *versus* ECX 2/79 (2.5 per cent); *P* = 0.123) or after oesophageal resections (FLOT 1/121 (0.8 per cent) *versus* ECX 3/121 (2.5 per cent); *P* = 0.368), open and (FLOT 3/121 (2.5 per cent) *versus* ECX 2/121 (1.7 per cent); *P* = 0.555) minimally invasive. There was no statistically significant difference in oesophageal–enteric leaks between the FLOT group (4/100 (4 per cent)) and the ECF/ECX group (7/100 (7 per cent; *P* = 0.352)). The overall rate of 30-day return to theatre rate was lower in the FLOT cohort (0 (0 per cent)) than in the ECX cohort (10 (10 per cent; *P* = 0.001). However, there was no difference in 30-day return to theatre specifically for anastomotic leaks: 0 (0 per cent) *versus* three (3 per cent; *P* = 0.081). Non-leak-related return to the theatre was more common in the ECF/ECX group (six (6 per cent); *P* = 0.013) (*Tables S4* and *S5*).

Morbidities were comparable in both groups, with no significant difference across all systemic complications (*Table 3*). There was no difference in the overall Clavien-Dindo classification of complications in those severe complications graded IIIb and above (32 per cent *versus* 33 per cent; *P* = 0.880).

**Table 3:** Short-term perioperative outcomes of the FLOT and ECF/ECX cohorts (a binary logistic regression was performed adjusting for sex, age, BMI, and ASA status)

Variable		FLOT	ECF/ECX	OR	*P*
**Operation (oesophageal)**	Open (Ivor Lewis/thoracoabdominal)	12 (6)	37 (18)		< 0.001
	Minimally invasive Ivor Lewis (robotic/laparoscopic)	52 (26)	26 (13)		< 0.001
**Operation (gastric)**	Total gastrectomy	17 (8)	17 (8)		0.849
	Subtotal gastrectomy	19 (9)	20 (10)		0.718
**Gastrointestinal**	Oesophagoenteric leak	4 (4)	7 (7)		0.281
	Ileus	3 (3)	3 (3)		
	Small bowel obstruction	0 (0)	3 (3)		
	Feeding tube complication	0 (0)	2 (2)		
	Pyloroplasty complication	1 (1)	1 (1)		
	*Clostridium difficile* infection	4 (4)	2 (2)		
	Pancreatitis	1 (1)	0 (0)		
	Gastrointestinal bleeding	2 (2)	2 (2)		
	Delayed gastric emptying	3 (3)	10 (10)		
	Total	18 (18)	30 (30)		0.088
**Pulmonary**	Pneumonia	39 (39)	39 (39)		0.869
	Pleural effusion	15 (15)	16 (16)		
	Pneumothorax	5 (5)	2 (2)		
	Respiratory failure	6 (6)	4 (4)		
	Acute aspiration	2 (2)	3 (3)		
	Tracheobronchial injury	0 (0)	1 (1)		
	Total	46 (46)	45 (45)	1.025	0.937
**Cardiac**	Atrial dysrhythmia	7 (7)	13 (13)		0.303
	Cardiac arrest	1 (1)	0 (0)		
	Ventricular dysrhythmia	1 (1)	0 (0)		
	Congestive heart failure	1 (1)	0 (0)		
	Total	10 (10)	13 (13)	1.194	0.718
**Urology**	Acute renal insufficiency	1 (1)	4 (4)		0.534
	UTI	2 (2)	3 (3)		
	Urinary retention	4 (4)	5 (5)		
	Total	7 (7)	10 (10)	1.102	0.860
**Neurology**	Recurrent nerve injury	0 (0)	1 (1)		0.800
	Other neurological injury	1 (1)	1 (1)		
	Acute delirium	4 (4)	5 (5)		
	Total	5 (5)	6 (6)	1.111	0.871
**Infection**	Wound	3 (3)	7 (7)		0.529
	Intrathoracic/intrabdominal sepsis	3 (3)	5 (5)		
	Generalized sepsis	9 (9)	7 (7)		
	Other infections	1 (1)	3 (3)		
	Total	16 (16)	19 (19)	1.319	0.485
**Thromboembolic**	DVT	3 (3)	1 (1)		0.614
	PE	1 (1)	2 (2)		
	Stroke	1 (1)	2 (2)		
	Thrombophlebitis	0 (0)	1 (1)		
	Total	5 (5)	6 (6)	1.179	0.810
**Wound**	Thoracic/abdominal wound dehiscence	0 (0)	2 (2)		0.218
	Acute diaphragmatic hernia	0 (0)	1 (1)		
	Total	0 (0)	3 (3)	1.404	0.081
**Other**	Chyle leak	3 (3)	1 (1)		0.043
	Reoperation for other reason	0 (0)	5 (5)		
	Multiple organ dysfunction	0 (0)	2 (2)		
	Total	3 (3)	8 (8)	2.435	0.212

Data are presented as *n* (%). FLOT, fluorouracil plus leucovorin, oxaliplatin, and docetaxel chemotherapy; ECF/ECX, (epirubicin, cisplatin, capecitabine (X) or 5-fluorouracil (F)). UTI, urinary tract infection; DVT, deep vein thrombosis; PE, pulmonary embolism.

The 30- and 90-day mortality rates were comparable between both cohorts (FLOT 0/100 (0 per cent) *versus* ECX 3/100 (3 per cent); *P* = 0.081)) and (FLOT 0/100 (0 per cent) *versus* 2/100 (2 per cent); *P* = 0.155)).

### Secondary analysis

A mean propensity score was calculated for both groups and was relatively similar at 0.598 within the FLOT group *versus* 0.618 in the ECX group. When considering the highest 30 matched patients, there were no significant differences in anastomotic leaks (FLOT 2/30; ECX 3/30 (*P* = 0.640)), 30-day reoperations (FLOT 0/30; ECX 3/30 (*P* = 0.076)), 30-day mortality (FLOT 0/30; ECX 2/30; *P* = 0.150), and length of stay (FLOT median 13 days *versus* ECX median 15 days; *P* = 0.275) (*Table S6*).

## Discussion

Following the publication of the Medical Research Council Adjuvant Gastric Infusional Chemotherapy (MAGIC), perioperative chemotherapy became a standard of care for patients with resectable gastric and oesophageal cancer^8^. A significant challenge in the perioperative setting of patients with resectable disease is maintaining a balance between the efficacy of neoadjuvant therapy and acceptable toxicity.

In the FLOT4 trial, patients had a higher rate of curative surgery and improved median overall survival (50 *versus* 35 months) *versus* ECF/ECX, without an increase in surgical morbidity and mortality, reoperations, and hospitalization time. As a result, many centres have adopted the FLOT perioperative regimen as the current standard of care.

Despite this, evidence remains sparse about the short and medium-term perioperative morbidity associated with FLOT, especially in the poorer performance status groups. While studies have shown this to be relatively safe, increased chemotherapy-related toxicity and higher risks of complications have been reported in the elderly with resectable disease^16^. There is limited evidence regarding surgical perioperative outcomes related to FLOT *versus* other regimens.

In the present study, the cohorts receiving FLOT and ECF/ECX were mostly patients over the age of 60 years with a BMI between 20 and 30 kg/m^2^ and a WHO performance status of 1. Other than an increase in minimally invasive procedures for oesophageal cancer, the two patient groups were comparable and had similar outcomes. Hospital length of stay was slightly longer for patients having open surgery *versus* those undergoing a minimally invasive procedure (median open 15 days *versus* minimally invasive 12 days; *P* = 0.035).

These institutional results support the perioperative morbidity results of the FLOT4 trial. There were no significant differences in perioperative complications between cohorts across all systemic classifications of benchmark complications on primary or secondary (propensity-matched) analysis^14^. There were no differences in outcome with regard to OG leaks, either when separately analysed by cancer pathology (gastric (FLOT 2.4 per cent *versus* ECX 3.3 per cent; *P* = 0.727) or oesophageal (FLOT 2.4 per cent *versus* 4.4 per cent; *P* = 0.474)) or surgical approach (open (FLOT 0 per cent *versus* 2 per cent; *P* = 0.155) *versus* minimally invasive (FLOT 2 per cent *versus* ECX 2 per cent; *P* = 0.999)). There was also no statistical difference in the return to theatre due to anastomotic leak rate (FLOT 0 per cent *versus* 3.0 per cent; *P* = 0.081), or in 30- (FLOT 0 per cent *versus* 3.0 per cent; *P* = 0.081) or 90-day (FLOT 0 per cent *versus* 2.0 per cent; *P* = 0.155) mortality.

FLOT has been reported to induce significantly higher pathological complete regression rates than ECF/ECX (20/128 (16 per cent) *versus* 8/137 (6 per cent); *P* = 0.02)^10^. While not the focus of the present study, the results support this previous finding. A significantly higher rate of complete tumour pathological response was seen in the FLOT group (20/100 (20 per cent) *versus* 10/100 (5 per cent); *P* = 0.048), and circumferential margin positivity was also lower in the FLOT group (5/100 (5 per cent) *versus* 14/100 (14 per cent); *P* = 0.03) (*Table 2*).

The study is limited due to the use of a consecutive case series from a single centre, with no prospective evaluation of surgical quality among the operating surgeons. The study is prone to historical bias due to the adoption of minimally invasive oesophagectomy and enhanced recovery pathways during the study period. These modifications may have influenced the length of stay. However, the results support the use of the perioperative FLOT regimen as the standard of care for locally advanced resectable OG cancer.

## Supplementary Material

zrac003_Supplementary_Data
