# Supplementary material for: Effect of perioperative FLOT versus ECF/ECX on short-term outcomes after surgery for resectable oesophagogastric adenocarcinoma: propensity score-matched study
Source: BJS Open. 2022 Feb 23;6(1):zrac003. doi: 10.1093/bjsopen/zrac003 (PMC8864466; doi:10.1093/bjsopen/zrac003)
Supplement: zrac003_Supplementary_Data [file zrac003_supplementary_data.zip › FINAL Supplementary Tables.docx]

|  | 0 | I | IIA | IIB | III | IV | p |
| --- | --- | --- | --- | --- | --- | --- | --- |
| FLOT | 3 | 9 | 1 | 7 | 12 | 1 | 0.426 |
| ECX | 0 | 7 | 0 | 9 | 9 | 0 |  |

Table S1. Gastric pathological staging between FLOT vs ECX peri-operative cohorts

|  | 0 | I | IIA | IIB | III | IVA | p |
| --- | --- | --- | --- | --- | --- | --- | --- |
| FLOT | 13 | 8 | 4 | 5 | 26 | 11 | 0.070 |
| ECX | 3 | 6 | 6 | 9 | 33 | 18 |  |

Table S2. Oesophageal pathological staging between FLOT vs ECX peri-operative cohorts

|  |  | Minimum | Maximum | Median | Interquartile percentile (25-75) | p |
| --- | --- | --- | --- | --- | --- | --- |
| BMI (Kg/m2) | FLOT | 17.3 | 40.30 | 25.65 | 23-29.4 | 0.451 |
|  | ECX | 19.7 | 42.90 | 26.35 | 23.7-28.9 |  |
| Age | FLOT | 24 | 81 | 65.5 | 53.3-70 | 0.362 |
|  | ECX | 28 | 82 | 64 | 57.3-72 |  |
| Length of stay (days) | FLOT | 5 | 129 | 12 | 10-15 | 0.035 |
|  | ECX | 2 | 86 | 15 | 10-23 |  |
| Follow up (months) | FLOT | 0.0 | 19 | 4 | 1-9 | 0.001 |
|  | ECX | 0.0 | 71 | 28 | 13-37.5 |  |
| Positive LN | FLOT | 0 | 34 | 0 | 0-2 | 0.377 |
|  | ECX | 0 | 18 | 0 | 0-2 |  |
| Harvested LN | FLOT | 11 | 83 | 33 | 23-43 | 0.262 |
|  | ECX | 9 | 88 | 36 | 26-45 |  |

Table S3. Baseline descriptive measures of spread of both FLOT vs ECX. Comparison was made between both cohorts using a Mann-Whitney U Test.

|  |  | FLOT | | ECX | | OR | p |
| --- | --- | --- | --- | --- | --- | --- | --- |
| Variable |  | Frequency | Percentage | Frequency | Percentage |  |  |
| Cumulative complication  number | 1 | 33 | 53.2 | 29 | 46.8 |  | 0.534 |
|  | 2 | 16 | 45.7 | 19 | 54.3 |  |  |
|  | 3 | 6 | 40 | 9 | 60 |  |  |
|  | 4 | 1 | 20 | 4 | 80 |  |  |
|  | 5 | 1 | 100 | 0 | 0 |  |  |
| Anastomotic leak |  | 4 | 4.0 | 7 | 7.0 | 1.579 | 0.490 |
| Chyle leak |  | 3 | 3.0 | 1 | 1.0 | 0.260 | 0.254 |
| CCU readmission |  | 4 | 4.0 | 6 | 6.0 | 1.520 | 0.550 |
| Reintubation |  | 5 | 6.0 | 11 | 12.2 | 2.062 | 0.213 |
| 30 day mortality |  | 0 | 0.0 | 3 | 3.0 |  | 0.081 |
| 30-90 day mortality |  | 0 | 0.0 | 2 | 2.0 |  | 0.155 |

Table S4. Short term peri-operative outcomes of peri-operative chemotherapy cohort and binary logistic regression of outcomes adjusting for gender, age category, BMI category and ASA.

|  | | FLOT | | ECX | | p |
| --- | --- | --- | --- | --- | --- | --- |
| Variable | | Frequency | Percentage | Frequency | Percentage |  |
| Anastomotic leak |  | 4 | 4.0 | 7 | 7.0 | 0.352 |
| Gastric |  | 0 | 0.0 | 2 | 2.5 | 0.123 |
| Oesophageal |  | 4 | 3.3 | 5 | 4.1 | 0.868 |
|  | Open | 1 | 0.8 | 3 | 2.5 | 0.368 |
|  | Min inv | 3 | 2.5 | 2 | 1.7 | 0.555 |
| 30-day reoperation (All) | 10 (5.0%) | 0 | 0.0 | 10 | 5.0 | 0.001 |
| 30-day reoperation (leak related) | 3 (1.5%) | 0 | 0.0 | 3 | 1.5 | 0.081 |
| 30-day reoperation (non-leak related) | 6 (3.0%) | 0 | 0.0 | 6 | 3.0 | 0.013 |

Table S5. Comparison of anastomotic leaks and reoperations in both cohorts (FLOT vs ECX).

|  |  | FLOT | | | ECX | | | p |
| --- | --- | --- | --- | --- | --- | --- | --- | --- |
|  |  | Count | Mean | Median | Count | Mean | Median |  |
| Gender | Male | 27 |  |  | 27 |  |  | ns |
|  | Female | 3 |  |  | 3 |  |  |  |
| ASA | ASA 1+2 | 19 |  |  | 21 |  |  | 0.584 |
|  | ASA 3+4 | 11 |  |  | 9 |  |  |  |
| Tumour site | Oesophageal | 27 |  |  | 24 |  |  | 0.278 |
|  | Gastric | 3 |  |  | 6 |  |  |  |
| Age |  |  | 74 |  |  | 77 |  | 0.331 |
| BMI |  |  | 27.04 |  |  | 28.03 |  | 0.234 |
| Anastomotic Leak | No | 28 |  |  | 27 |  |  | 0.640 |
|  | Yes | 2 |  |  | 3 |  |  |  |
| Reop-30 | No | 30 |  |  | 27 |  |  | 0.076 |
|  | Yes | 0 |  |  | 3 |  |  |  |
| Mortality 30 | No | 30 |  |  | 28 |  |  | 0.150 |
|  | Yes | 0 |  |  | 2 |  |  |  |
| Mortality 90 | No | 30 |  |  | 30 |  |  | ns |
|  | Yes | 0 |  |  | 0 |  |  |  |
| LOS |  |  | 14 | 13 |  | 16 | 15 | 0.275 |

Table S6. A propensity match was undertaken for validation. A propensity analysis of the highest matched 30 FLOT vs ECX patients displays similar non-significant outcome between cohorts.
