# Supplementary material for: Effect of perioperative FLOT versus ECF/ECX on short-term outcomes after surgery for resectable oesophagogastric adenocarcinoma: propensity score-matched study
Source: BJS Open. 2022 Feb 23;6(1):zrac003. doi: 10.1093/bjsopen/zrac003 (PMC8864466; doi:10.1093/bjsopen/zrac003)
Supplement: zrac003_Supplementary_Data [file zrac003_supplementary_data.zip › Supplementary Table Order BJS open - February 2022.docx]

Supplementary Table 1. Gastric pathological staging between FLOT vs ECX peri-operative cohorts.

Supplementary Table 2. Oesophageal pathological staging between FLOT vs ECX peri-operative cohorts

Supplementary Table 3. Baseline descriptive measures of spread of both FLOT vs ECX. Comparison was made between both cohorts using a Mann-Whitney U Test.

Supplementary Table 4 – Short term peri-operative outcomes of peri-operative chemotherapy cohort and binary logistic regression of outcomes adjusting for gender, age category, BMI category and ASA.

Supplementary Table 5. Comparison of anastomotic leaks and reoperations in both cohorts (FLOT vs ECX).

Supplementary table 6. A propensity match was undertaken for validation. A propensity analysis of the highest matched 30 FLOT vs ECX patients displays similar non-significant outcome between cohorts.
